# 

**Published:** 1909-06-15

**Authors:** 


CONTENTS

Event and Comment - - - - - - - - 281
Silicious Cement - - - - *By W. V-B. Ames, D.D.S.* 283
"Artificial Enamel" - - *By Harry L. Belcher, D.D.S.* 309
The Probable Usefulness of the Silicate Cements, *By W. B. Dunning, D.D.S.* 312
Indents - - - - - - - - - - - - 323
Announcements - - - - - - - - - 331

